# Transcriptomic characterization of *Caecomyces churrovis*: a novel, non-rhizoid-forming lignocellulolytic anaerobic fungus

**DOI:** 10.1186/s13068-017-0997-4

**Published:** 2017-12-20

**Authors:** John K. Henske, Sean P. Gilmore, Doriv Knop, Francis J. Cunningham, Jessica A. Sexton, Chuck R. Smallwood, Vaithiyalingam Shutthanandan, James E. Evans, Michael K. Theodorou, Michelle A. O’Malley

**Affiliations:** 1 Department of Chemical Engineering, University of California, Santa Barbara, CA 93106 USA; 2 Environmental Molecular Sciences Laboratory, Pacific Northwest National Laboratory, Richland, WA 99354 USA; 3 Agriculture Centre for Sustainable Energy Systems (ACSES), Animal Production, Welfare and Veterinary Sciences, Harper Adams University, Newport, Shropshire TF10 8NB UK

**Keywords:** Anaerobic fungi, Neocallimastigomycota, Cellulase, Enzyme, Cellulosome

## Abstract

Anaerobic gut fungi are the primary colonizers of plant material in the rumen microbiome, but are poorly studied due to a lack of characterized isolates. While most genera of gut fungi form extensive rhizoidal networks, which likely participate in mechanical disruption of plant cell walls, fungi within the *Caecomyces* genus do not possess these rhizoids. Here, we describe a novel fungal isolate, *Caecomyces churrovis*, which forms spherical sporangia with a limited rhizoidal network yet secretes a diverse set of carbohydrate active enzymes (CAZymes) for plant cell wall hydrolysis. Despite lacking an extensive rhizoidal system, *C. churrovis* is capable of growth on fibrous substrates like switchgrass, reed canary grass, and corn stover, although faster growth is observed on soluble sugars. Gut fungi have been shown to use enzyme complexes (fungal cellulosomes) in which CAZymes bind to non-catalytic scaffoldins to improve biomass degradation efficiency. However, transcriptomic analysis and enzyme activity assays reveal that *C. churrovis* relies more on free enzymes compared to other gut fungal isolates. Only 15% of CAZyme transcripts contain non-catalytic dockerin domains in *C. churrovis*, compared to 30% in rhizoid-forming fungi. Furthermore, *C. churrovis* is enriched in GH43 enzymes that provide complementary hemicellulose degrading activities, suggesting that a wider variety of these activities are required to degrade plant biomass in the absence of an extensive fungal rhizoid network. Overall, molecular characterization of a non-rhizoid-forming anaerobic fungus fills a gap in understanding the roles of CAZyme abundance and associated degradation mechanisms during lignocellulose breakdown within the rumen microbiome.

**Electronic supplementary material:**

The online version of this article (10.1186/s13068-017-0997-4) contains supplementary material, which is available to authorized users.

## Background

Anaerobic gut fungi are robust degraders of plant biomass in the guts of ruminants and other large monogastric herbivorous mammals [1]. They have also been identified using microscopy and molecular methodologies in the digestive tract of herbivorous reptiles [2]. Due to the large amount of biomass-degrading enzymes that these organisms secrete [3–5], they have potential for application in the production of lignocellulose-derived chemical products [6]. Most known genera (*Anaeromyces*, *Buwchfawromyces*, *Neocallimastix*, *Oontomyces*, *Orpinomyces*, *Pecoramyces*, *Piromyces*) of gut fungi have an extensive network of penetrating rhizoids (tapering mycelia) that aid, alongside enzymatic activity, in biomass colonization and deconstruction [7–11]. However, two known genera within the clade of anaerobic fungi (*Caecomyces*, *Cyllamyces*) do not produce rhizoidal networks, but form a limited system simply capable of attaching to plant biomass [7, 12]. However, like their rhizoidal counterparts, non-rhizoid producing gut fungi are proficient degraders of crude plant biomass. This raises the possibility that there are differences between the diversity of enzymes employed by rhizoid-forming vs. non-rhizoid-forming fungi, and/or the mechanisms they use for enzymatic degradation of lignocellulose.

While there is a general lack of genomic information for the Neocallimastigomycota, recently five complete genomes and transcriptomes have been published for rhizoid-forming anaerobic fungi—two representatives from the *Piromyces* genus, one each from *Anaeromyces* and *Neocallimastix* [13], and one from *Orpinomyces* [14] (recently reclassified as *Pecoramyces* [10]). Interestingly, this wealth of sequencing data has revealed that anaerobic fungi can draw from two modes of biomass-degradation via the secretion of freely diffusive enzymes as well as via fungal cellulosomes (complexes of enzymes tethered together for synergistic action) [13]. However, at the present time, no high-resolution transcriptomic or genomic information has been reported for non-rhizoid-forming isolates. This precludes insight into the enzymatic machinery of non-rhizoid-forming fungi, or the mode of biomass degradation that they favor.

Here, we describe a novel species of non-rhizoid-forming fungi belonging to the *Caecomyces* genus isolated from the fecal pellets of a Navajo Churro sheep collected in 2015. While other *Caecomyces* isolates have been described using morphological and phylogenetic analyses [15, 16], including some analysis of the cellulolytic enzyme activity [17], extensive genomic or transcriptomic sequencing has not been completed. By assembling the first sequenced transcriptome for an anaerobic gut fungus within the *Caecomyces* genus, *C. churrovis*, our analysis enabled us to identify the range of CAZymes available to a non-rhizoidal genus and test the null hypothesis that additional degradation mechanisms, mechanical or enzymatic, are not required for dissolution of plant biomass. This isolated fungal strain was assessed for its ability to grow on a range of substrates, and demonstrated a greater preference for soluble substrates compared to other rhizoid-forming strains that have been analyzed to date [5]. Transcriptome assembly and analysis identified a broad range of CAZymes within the genome, including a relative abundance of carbohydrate esterase and hemicellulase (GH 11/12, 43) transcripts. Comparison to other sequenced gut fungal isolates also revealed a greater reliance on free enzymes rather than enzymes bound in fungal cellulosome complexes.

## Results and discussion

### Isolation and molecular classification of *C. churrovis*

**Fig. 1:**
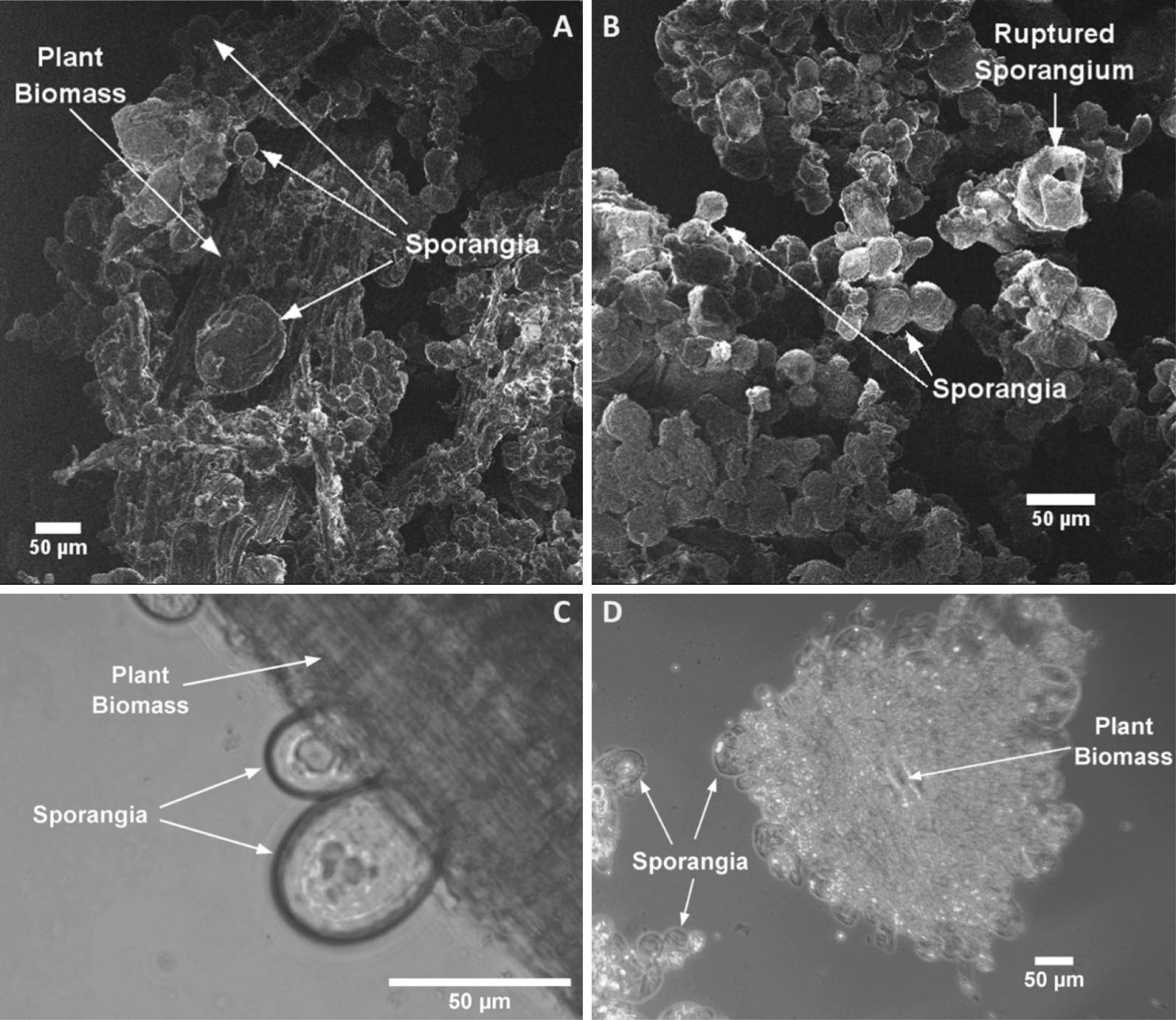
*C. churrovis* cultured on reed canary grass covers plant biomass surface in the absence of a rhizoid network. Helium ion microscopy (**A**, **B**) of *C. churrovis* grown on crude plant material (reed canary grass) highlights the spherical sporangia and lack of extensive mycelial network. The fungus shows a wide range of size of sporangia, likely due to different phases of the growth cycle. In **A**, **C**, the reed canary grass is visible and *C. churrovis* sporangia are attached to it via small “holdfasts.” Images **B**, **D** show a small particle of plant material completely covered in sporangia. Image B also shows a ruptured sporangium that has broken open to let out the motile zoospores as part of the gut fungal reproductive cycle

*Caecomyces churrovis* was isolated from the feces of a Navajo Churro sheep at the Santa Barbara Zoo. Microscopic analysis suggested that *C. churrovis* is a monocentric fungus, and confirmed that it does not possess an extensive rhizoidal network to penetrate biomass. Rather, *C. churrovis* forms a large, spherical sporangium with holdfast structures to attach it to plant biomass and other solid substrates (Fig. 1). Although there is not an extensive rhizoidal network that aids in biomass disruption, these fungi still localize to, and colonize, the cellulose-rich surface of plant biomass. Figure 1 shows sections of plant material almost entirely covered in *C. churrovis* sporangia. Sporangia are present within a range of sizes, some near 20 µm in diameter, while others are greater than 50 µm in diameter. Furthermore, Fig. 1B highlights the lytic life cycle of gut fungi, as a fraction of colonized mature zoosporangia rupture and collapse over time, releasing the cellular contents and motile zoospores. These zoospores likely chemotax towards a carbon source (e.g., biomass) and initiate the formation of new monocentric sporangia.

**Fig. 2:**
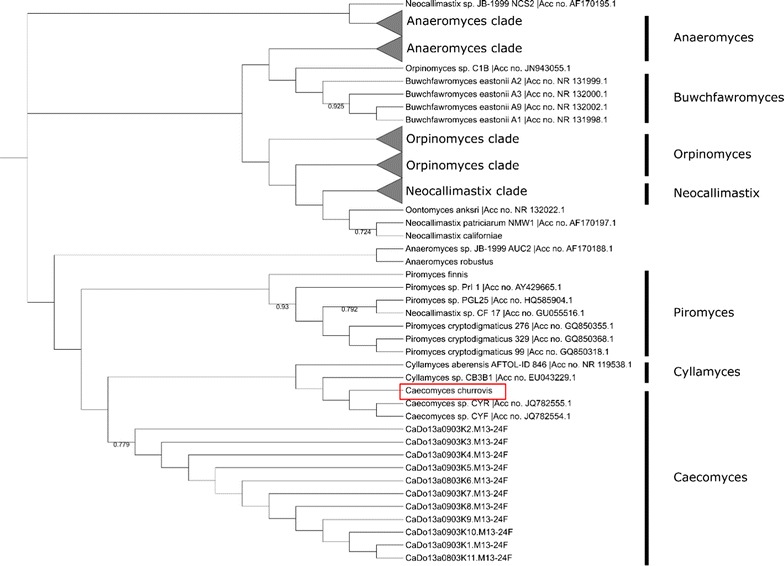
ITS1 phylogenetic analysis clusters *C. churrovis* with *Caecomyces* strains. ClustalW alignment and maximum parsimony phylogenetic analysis bootstrapped with 1000 replications was used to perform phylogenetic analysis. Among the sequences used in the alignment all genera of anaerobic gut fungi are represented and *C. churrovis* clusters exclusively with *Caecomyces* strains

While morphological characterization indicates that the isolated fungal strain is likely a member of the *Caecomyces* genus, anaerobic fungi are often pleomorphic and require phylogenetic analysis of conserved genomic sequences to confirm classification. The internal transcribed spacer (ITS) regions are commonly used to determine the genera of newly isolated fungi [18–21]. The ITS1 and ITS2 regions for *C. churrovis* (GenBank #MF460993) were amplified and sequenced using PCR primers JB206 and JB205 [20], and subjected to phylogenetic analysis. Given the abundance of ITS1 sequences deposited in GenBank, we relied primarily on comparative alignment with this region for preliminary identification and classification. While other neighboring DNA sequences, such as the ITS2 [20] and large subunit (28S) [22], have been used for phylogenetic analysis, restricting our analysis to ITS1 enabled comparison with the maximum number of GenBank submissions. From this analysis (Fig. 2), the isolated strain *C. churrovis* clearly clusters with other *Caecomyces* fungi. Subsequently, additional phylogenetic analysis was completed using only sequences from fungi within the *Caecomyces* genus to ensure that this isolated strain was significantly divergent from previously characterized strains to constitute its classification as a novel species. Generation of the phylogenetic tree isolated *C. churrovis* from other *Caecomyces* strains with a bootstrap value of 98, indicating that this node occurred in 98% of trees generated during the bootstrap analysis (Additional file 1: Figure S1). Thus, the ITS1 region of *C. churrovis* was significantly different compared to other *Caecomyces* strains with ITS1 sequences available on GenBank, suggesting that it represents a novel strain. This finding was confirmed by performing a phylogenetic analysis on the full ITS sequence (Additional file 1: Figure S2).

### *C. churrovis* is capable of growth on crude biomass and fermentable sugars

**Fig. 3:**
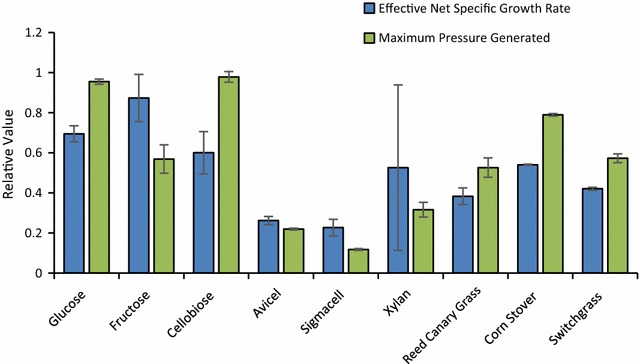
*C. churrovis* grows faster on soluble substrates than crude biomass. When grown on simple sugars, *C. churrovis* yielded the largest effective net-specific growth rates and the greatest overall production of fermentation gases (shown as maximum accumulated pressure). On biomass substrates, effective net-specific growth rates were smaller, with a reduction in the total pressure generated from fermentation gases. Error bars represent standard deviation of three biological replicates in each case

The rate and extent of growth of *C. churrovis* on a range of soluble and polymeric carbohydrates, including native plant biomass, was determined. Substrates included mono- and di-saccharides (glucose, fructose, arabinose, xylose, mannose, cellobiose, maltose, sucrose), polysaccharides (cellulose: Avicel, Sigmacell; hemicellulose: xylan from corn stover), and plant biomass (reed canary grass, corn stover, alfalfa stems, switchgrass). Due to the nature of fungal growth and the use of insoluble carbon sources, accumulation of fermentation gas pressure in the sealed culture tubes is commonly used to measure growth [23, 24]. Effective net-specific growth rates were determined by calculating the slope of a log-linear plot of accumulated pressure versus time during exponential pressure generation (exponential growth phase). This analysis identified a preference for *C. churrovis* growth on simple sugars compared to complex biomass. Effective net-specific growth rates of 0.050 ± 0.002 h^−1^ and 0.063 ± 0.008 h^−1^ were calculated for glucose and fructose, respectively, while those calculated for polymeric substrates ranged from 0.028 ± 0.003 h^−1^ for growth on reed canary grass to 0.039 ± 0.0002 h^−1^ for growth on corn stover (Fig. 3).

Among soluble sugars, the highest overall pressure production was observed during growth on cellobiose (15.37 ± 0.42 psig) and glucose (15.0 ± 0.20 psig), with lower pressure observed during growth on fructose (8.93 ± 1.1 psig) suggesting that greater growth and metabolic activity occurs on glucose and cellobiose (Fig. 3). This may be related to regulation mechanisms behind the production of biomass-degrading enzymes, as glucose has been shown to function as a carbon catabolite repressor for CAZymes [5], resulting in fewer cellular resources being diverted to produce enzymes that are not necessary for growth on these substrates. Fungal growth was not observed on the five-carbon sugars xylose and arabinose, or six-carbon sugars galactose and mannose, despite the presence of these sugars in plant cell walls. Cellobiose—a breakout product of cellulose—was the only disaccharide that supported growth, as no growth was observed on maltose and sucrose.

For polymeric substrates, minimal growth was detected on purified crystalline cellulose (Avicel^®^ and Sigmacell) with total accumulated pressure only 1.2–3.5 psig greater than total accumulated pressure of blank cultures in the absence of a carbon source. However, the lack of growth on crystalline cellulose may be linked to the lack of rhizoids produced by *C. churrovis*. The small particle size (50 µm) of these crystalline cellulose substrates compared to milled plant biomass (4 mm) may lead to more dense substrate packing, preventing access of the gut fungus to the cellulose beyond the top surface layer. In contrast, rhizoidal networks produced by other gut fungi likely penetrate the cellulose, disrupting the packing and improving access of zoospores and secreted enzymes to exposed cellulose chains. *C. churrovis* growth was observed on xylan for some cultures, but was inconsistent and resulted in large error in growth rate measurements (Fig. 3). Growth rates observed on plant biomass substrates varied significantly with net-specific growth rates on corn stover (0.039 ± 0.0002 h^−1^) significantly higher (*P* = 1.4 × 10^−4^) than on switchgrass and reed canary grass (0.030 ± 0.0005 h^−1^ and 0.028 ± 0.003 h^−1^, respectively), and no growth observed on alfalfa stems. This observation is consistent with the cell wall composition of alfalfa stems, which have a greater relative pectin content compared to the other grasses [25], which may hinder fungal growth. The greatest pressure was observed during growth on corn stover (12.4 ± 0.10 psig), while lower total pressure was measured on reed canary grass and switchgrass (8.27 ± 0.76 and 9.0 ± 0.35 psig, respectively). This suggests that differing composition of the plant material in terms of lignin content and sugar composition [25, 26] may impact growth of *C. churrovis*. Corn stover comprises 32–36% glucan [26] while reed canary grass and switch grass comprise 20.9–26.5 and 27.3–32.2% cellulose, respectively [25]. This greater glucan concentration in corn stover may have resulted in greater maximum pressure accumulation during growth on this substrate.

### Comparative transcriptomic analysis of *C. churrovis* against rhizoid-forming gut fungi

To probe deeper into the biomass-degrading capacity of *C. churrovis*, the transcriptome was analyzed to identify its putative array of biomass-degrading enzymes. A full transcriptome was assembled for *C. churrovis* by pooling RNA from batch cultures grown on glucose, cellobiose, cellulose, and reed canary grass to obtain an inclusive set of expressed genes under these varied growth conditions. The transcriptome was sequenced in a strand-specific manner on an Illumina NextSeq and assembled de novo using the Trinity algorithm [27], previously used for assembly of anaerobic fungal transcriptomes in lieu of genomic information [5]. The assembled transcriptome contained 36,595 transcripts, representing a predicted 33,437 genes (Table 1). Comparatively, the transcriptome of *Piromyces finnis* assembled using Trinity comprises 27,140 transcripts and 22,959 predicted genes, while the transcriptomes of *Anaeromyces robustus* and *Neocallimastix californiae* assembled using Rnnotator [28] comprise 17,127 and 29,649 transcripts, and 16,038 and 27,671 predicted genes, respectively [5]. The number of genes identified by transcriptomes of each of these fungi is significantly higher than the number of genes identified by their sequenced genomes [13]. However, comparisons between transcriptomes are more appropriate than comparisons of the *C. churrovis* transcriptome to the genomes of previously sequenced gut fungi. The *C. churrovis* transcriptome was functionally annotated using a combination of alignments to the NCBI non-redundant protein database [29] and EMBL-EBI InterPro database [30]. This analysis resulted in only 9.33% of the transcriptome receiving annotation from BLAST alignments and 72.52% receiving protein domain annotations from InterProScan. The Blast2GO program was then used to assign gene ontology terms and enzyme commission (EC) numbers yielding 33.22 and 7.55% annotation, respectively (Table 1). The large amount of unannotated transcripts reflects a lack of both sequence availability and biochemical knowledge of the anaerobic gut fungi and other primitive clades compared to the higher fungi.

**Table 1:** *C. churrovis* transcriptome sequencing and annotation summary

Transcriptome size (bp)	30,884,864
Number of transcripts	36,595
Average length (bp)	843
Number of predicted genes (transcripts less isoforms)	33,437
Number of clusters	116,890,119
Number of reads	233,780,238
Read length	75
Coverage	567.7×
% with EC number	7.55%
% with BLAST hits	9.33%
% with gene ontology	33.22%
% with InterPro scan	72.52%

The transcriptome of *C. churrovis* was also aligned and compared to the transcriptomes of the rhizoid-forming fungi *Anaeromyces robustus* (IF 551676), *Neocallimastix californiae* (IF 551675), and *Piromyces finnis* (IF 551677) [5] using a nucleotide blast (blastn). Due to the unique nucleotide composition of gut fungal genes [31, 32], nucleotide blast was used to probe the gene similarity at nucleotide sequence rather than amino acid sequence level. This analysis resulted in alignment of 13,155 transcripts (35.95%) from the *C. churrovis* transcriptome to 7247 transcripts (42.31%) from *A. robustus*; 13,532 transcripts (36.98%) aligned to 11,100 transcripts (37.44%) from *N. californiae*; and 11,597 (31.69%) aligned to 6843 transcripts (40.23%) in *P. finnis* (Table 2). Thus, the *C. churrovis* transcriptome was less than 37% similar to each of these previously sequenced anaerobic fungi. This is similar to the results when *A. robustus*, *N. californiae*, and *P. finnis* are aligned to each other, with percent of homologous transcripts ranging from 36 to 49% (Additional file 1: Table S1). Those transcripts that were aligned across these four fungal transcriptomes are likely to represent basic functions that are necessary and conserved across all gut fungi. Conversely, sequences that were not aligned across the transcriptomes may represent niche functions specific to the different strains, but could also comprise false isoforms and transcript fusions that were generated by the alignment algorithms.

**Table 2:** Transcriptomic Comparison of *C. churrovis* to other Anaerobic Gut Fungi

	*Anaeromyces robustus*	*Neocallimastix californiae*	*Piromyces finnis*
# *C. churrovis* transcripts aligned	13,155	13,532	11,597
% *C. churrovis* transcriptome	35.95	36.98	31.69
# transcripts matched	7247	11,100	6843
% transcriptome aligned to	42.31	37.44	40.23

Functional annotations (especially EC numbers) were used to identify complete sugar catabolic pathways for *C. churrovis* to establish the key substrates that it is able to assimilate from the environment (Additional file 1: Figure S3). This analysis identified a complete glycolysis pathway for catabolism of glucose as well as complete xylose and fructose catabolism pathways. However, in the xylose catabolic pathway, ribokinase was identified via BLAST annotations but not EC number. This indicates less confidence in the assignment of this function and may explain the lack of growth on xylose for this anaerobic gut fungus. Complete catabolic pathways were not found for several biomass-derived sugars, including mannose, sucrose, and arabinose, which was consistent with growth phenotypes observed. Galactose catabolism was identified as complete for α-d-galactose, but not β-d-galactose using NCBI Blast annotations in addition to EC numbers, although two enzymes were not present in the EC annotations. Incomplete pathways serve as an explanation for the lack of growth on these sugars in the growth studies that were performed. These results are similar to those produced in metabolic analysis of *A. robustus* and *N. californiae* [33], with missing enzymes for arabinose and galactose metabolism in both fungi and missing enzymes for sucrose and mannose metabolism in *A. robustus*. It is surprising, however, that several biomass-derived sugars were not catabolized by the anaerobic gut fungus despite the fact that they are predicted to be hydrolyzed by gut fungal enzymes (Table 3). Given the abundance of microorganisms in the rumen microbiome, many of these sugars are likely to be consumed by microbes other than the gut fungi. This may suggest that there is too much competition for these sugars in their native microbiomes and *C. churrovis* evolved to more efficiently catabolize glucose rather than compete for all plant-derived sugars. Thus, the role of the non-rhizoid-forming fungi in the rumen microbiome may be to primarily consume glucose and supply extra enzymes for biomass degradation for the more diverse microbial community.

**Table 3:** Comparison of cellulose machinery across four gut fungal strains

	Number of transcripts (# dockerin containing transcripts)^a^			
	*C. churrovis*	*N. californiae*	*A. robustus*	*P. finnis*
Hemicellulases				
GH11–12	63 (6)	67 (15)	30 (8)	35 (9)
GH11	60 (6)	59 (14)	30 (8)	31 (9)
GH43	59 (10)	35 (15)	16 (12)	11 (9)
GH10	15 (2)	67 (25)	15 (6)	16 (7)
GH39	3 (2)	9 (8)	4 (4)	1 (1)
GH30	2 (2)	2 (1)	2 (2)	1 (1)
Accessory enzymes				
Carbohydrate esterase	47 (1)	43 (7)	28 (3)	22 (5)
Pectin lyase	45 (2)	35 (0)	5 (0)	9 (0)
Polysaccharide deacetylase	42 (1)	93 (2)	58 (2)	48 (2)
Rhamnogalacturonate lyase	4 (3)	4 (1)	3 (1)	2 (1)
Pectinesterase	4 (0)	12 (0)	5 (0)	6 (0)
GH88	0 (0)	2 (2)	0 (0)	1 (1)
Cellulases				
GH9	29 (11)	25 (13)	15 (9)	12 (9)
GH6	27 (8)	22 (18)	5 (3)	8 (7)
GH45	26 (12)	24 (14)	13 (7)	11 (7)
GH48	25 (5)	24 (17)	7 (5)	14 (7)
GH1	20 (0)	16 (0)	11 (0)	9 (0)
GH5	19 (1)	48 (25)	22 (8)	27 (9)
GH3	16 (3)	33 (4)	16 (3)	12 (2)
GH16	3 (1)	15 (6)	9 (2)	5 (2)
GH8	2 (1)	4 (2)	1 (1)	1 (1)
GH31	1 (0)	7 (0)	6 (0)	1 (0)
Total	512 (77)	646 (189)	301 (84)	283 (89)

^a^Dockerin containing transcripts are expected to participate in cellulosome complex formation and the number shown in parentheses indicates the number of transcripts that contain at least one of these domains

### Comparison of CAZyme machinery reveals a dependency on free enzymes in *C. churrovis*

**Fig. 4:**
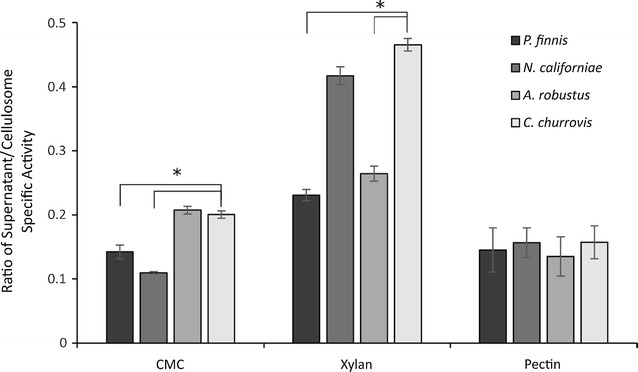
*C. churrovis* supernatant possesses higher relative activity than cellulose precipitated cellulosome compared to other fungi. Both culture supernatants and cellulose precipitated enzyme fractions were assessed for their activity on carboxymethyl cellulose, xylan, and pectin. Across the substrates tested, *C. churrovis* consistently had the highest specific activity in the supernatant compared to the cellulosome, including significantly (*P* < 0.05) more than *P. finnis* and *N. californiae* on CMC and significantly (*P* < 0.05) more than *P. finnis* and *A. robustus* on Xylan. These results suggest greater importance of free enzymes in *C. churrovis* and reflect the diverse array of enzymes possessed by anaerobic fungi. Protein gels are displayed in (Additional file 1: Figure S4). Error bars represent standard deviation of three replicates. *Represents significance, which was determined using the student’s t test

The functional protein annotations were also searched to identify CAZymes based on assignment of InterPro protein domains as an initial comparison. Priority was placed on identification of these enzymes, as they are critical for the degradation of plant material, a feature of anaerobic gut fungi that is of interest to exploit for bio-based fuel and chemical production. Our analysis identified 512 total CAZymes in *C. churrovis* (Table 3), including cellulases (GH1, GH3, GH5, GH6, GH8, GH9, GH16, GH31, GH45, GH48), hemicellulases (GH10, GH11, GH12, GH30, GH39, GH43), and other accessory enzymes (carbohydrate esterase, polysaccharide deacetylase, pectin lyase, pectinase, pectinesterase). Among the glycoside hydrolase (GH) transcripts, *C. churrovis* contains a much greater number of GH43 transcripts (11.5% of CAZymes) compared to the other fungal strains (3.8–5.4%). These enzymes are responsible for a variety of hemicellulose hydrolysis functions including xylosidase, arabinanase, arabinofuranosidase, and galactosidase activity [34]. This may indicate a greater emphasis on broad-ranging hemicellulose hydrolysis. While no fungal growth was detected on xylose, arabinose, and galactose, this extra activity may be necessary to break through hemicellulose in lieu of the targeted disruption from the rhizoids of the other fungi. In the transcriptomes of *N. californiae*, *A. robustus*, and *P. finnis*, GH5 represents the most abundant cellulase with 7.4, 7.3, and 9.5% of all CAZyme transcripts, respectively, but only 3.7% of all CAZyme transcripts in *C. churrovis* were members of this GH family. This family contains primarily endoglucanases and in *C. churrovis* the most abundant cellulase family, GH9, also primarily contains this activity. This may indicate a preference for this specific enzyme in the non-rhizoid-forming fungi, though it is unclear why this may be the case. The activity of *C. churrovis* cellulosomes and free enzymes on carboxymethyl cellulose (Fig. 4) is lower compared to the activity of the cellulosomes of both *N. californiae* and *P. finnis*, suggesting that these changes in enzyme preference may lead to changes in overall activity on recalcitrant substrates.

Interestingly, *C. churrovis* harbors fewer transcripts for polysaccharide deacetylases (8.2% of CAZymes) compared to the other rhizoid-forming fungi where it is the most abundant CAZyme family (14.3–19.2%). In contrast, the highest abundance accessory enzymes in *C. churrovis* were carbohydrate esterases (9.2% of CAZymes) containing SGNH hydrolase domains. A similar proportion of carbohydrate esterases was observed in *A. robustus*, but a smaller proportion was observed in *N. californiae* and *P. finnis*. These enzymes aid in the digestion of plant wall polysaccharides by removing acetylation and improving hydrolysis [35]. A greater proportion of the pectin lyase transcripts were also identified in *C. churrovis* (8.8% compared to 1.7–5.4%). The identification of many putative pectin degrading enzymes suggests that *C. churrovis* should be able to grow on pectin-rich grasses like alfalfa. However, this activity was not observed in *C. churrovis*, despite reports that rhizoid-forming fungi can subsist on alfalfa stems [5]. This growth discrepancy may be due to the fact that alfalfa stems formed a gelatinous layer on top of the plant material after autoclaving, forming an effective “barrier” against penetration by non-rhizoid-forming fungi like *Caecomyces*. It is therefore possible that this limited the ability of the fungus to access and colonize the plant material.

As a mechanism to more efficiently degrade plant biomass, anaerobic gut fungi have been described to form complexes of CAZymes (fungal cellulosomes, Additional file 1: Figure S3), bringing the activities of multiple enzymes in closer proximity to each other. Recently, structural scaffoldin proteins have been discovered that mediate this complex formation by binding to non-catalytic fungal dockerin domains that are distinct from previously described bacterial dockerins [13]. Given the similarity across gut fungal CAZymes, we hypothesized that some (or all) of this machinery might be shared between previously sequenced fungi and *C. churrovis*. Therefore, transcripts encoding for scaffoldin proteins in *C. churrovis* were identified by aligning the amino acid sequence of transcripts identified as scaffoldins in *P. finnis* [13] to the *C. churrovis* transcriptome using tblastn alignment. This revealed 38 transcripts with an alignment E-value of 0 (Additional file 1: Table S2), indicating that the scaffoldin machinery that forms fungal cellulosome complexes is actively transcribed under the substrate conditions encompassed in the transcriptome of *C. churrovis*. However, extensive Hidden Markov Model (HMM) analysis was not completed as done in previous work [13], largely due to the difficulties of applying such techniques in the absence of corresponding high-resolution genomes.

We also identified and compared the CAZyme transcripts containing non-catalytic fungal dockerin domains (NCDDs) (also referred to as CBM10s) to identify the protein components of the fungal cellulosome in *C. churrovis* (Table 3). While the general diversity of NCDD containing CAZyme transcripts was relatively consistent across strains with 45–55% cellulases, 36–47% hemicellulases, and 6–10% accessory enzymes, the percent of all CAZymes in *C. churrovis* with NCDDs was significantly lower compared to other strains. In *C. churrovis*, of the 512 CAZyme transcripts identified 77 also contained a fungal dockerin domain, representing 15% of all CAZyme transcripts. By comparison, the fraction of cellulosome-associated CAZymes in *C. churrovis* is much lower compared to rhizoid-forming *A. robustus*, *N. californiae*, and *P. finnis*, in which the dockerin-containing CAZyme transcripts represent 27.9, 29.3, and 31.4% of all CAZyme transcripts, respectively [5, 13]. This suggests that *C. churrovis* places greater emphasis on secreted un-complexed, free enzymes to attack plant biomass and release fermentable sugars compared to rhizoid-forming anaerobic fungi. *C. churrovis* does not maintain as much physical contact to the surface of plant material compared to rhizoid-forming strains that penetrate and intertwine with it. This dependence on free enzymes may maximize the area of the plant surface acted on by secreted CAZymes, especially if scaffoldin proteins are anchored to the fungal cell membrane or cell wall.

To test this finding, the activity of *C. churrovis* CAZymes on carboxymethyl cellulose (CMC), xylan, and pectin from cellulosome complexes was compared to the activity of all enzymes present in the fungal culture supernatants. Fungal cellulosomes were isolated through cellulose precipitation as previously described [5, 36]. This method enriches for cellulosome complexes rather than free enzymes. In contrast, the activity of the fungal supernatant contains cellulosome and free enzyme activities, in addition to non-cellulolytic proteins. Here, the enzyme-rich culture supernatant from *C. churrovis* possessed the highest activity relative to the prepared cellulosome compared to the other fungi tested (Fig. 4). Conversely, the cellulosome preparations of *P. finnis*, *N. californiae*, and *A. robustus* exhibited much greater activity than their corresponding culture supernatants containing a mixture of complexed and free enzymes. This finding is consistent with the hypothesis that *C. churrovis* is more dependent on free enzyme activity for the breakdown of cellulosic substrates compared to other rhizoid-forming genera of gut fungi that transcribe a higher fraction of cellulosome-associated enzymes. Furthermore, the activity on CMC, xylan, and pectin demonstrates the wide range of cellulolytic and hemicellulolytic enzyme activities as determined from transcriptomics.

## Conclusions

Among anaerobic gut fungi, the *Caecomyces* genus represents an interesting opportunity to identify the role of gut fungal enzymes in their native microbiome in the absence of extensive, invasive rhizoidal growth. Here, we have characterized the growth of the novel isolate, *C. churrovis* across plant biomass substrates ranging in complexity and composition. *C. churrovis* demonstrated the most rapid growth on free sugars like glucose, cellobiose, and fructose and no growth on other sugars that are derived from plant biopolymers. Despite the lack of invasive rhizoids, *C. churrovis* was capable of growth on complex plant biomasses reed canary grass, corn stover, and switchgrass. Sequencing and assembly of the first transcriptome for an anaerobic gut fungus within the *Caecomyces* genus identified a broad array of CAZymes, including an increased diversity of hemicellulases compared to its rhizoid-forming counterparts. Without the mechanical disruption provided by rhizoidal growth [11], the suite of enzymes secreted by *C. churrovis* was still sufficient for hydrolysis of crude plant material. Cellulosome complex forming scaffoldin proteins were identified in the transcriptome, but a smaller proportion of CAZyme transcripts containing NCDDs suggest a greater dependence on free enzymes for plant biomass degradation compared to rhizoid-forming gut fungal genera. Enzyme activity assays supported this hypotheses as *C. churrovis* cellulosome preparations showed the least improved biomass-degrading activity relative to fungal culture supernatants. Here, our study of a non-rhizoid-forming gut fungus highlights the capabilities of gut fungal enzymes as a mechanism for lignocellulose hydrolysis and, in the case of *C. churrovis*, a greater reliance on free enzymes.

## Methods

### Isolation and culture maintenance

Strictly anaerobic, aseptic techniques and an incubation temperature of 39 °C were used throughout for fungal isolation and culture maintenance. The headspace gas was 100% CO_2_ and the antibiotic, chloramphenicol, at a final medium concentration of 100 µg mL^−1^, was used in all liquid culture media, but not in agar containing roll tubes. *C. churrovis* (IF 553979) was isolated from fresh fecal pellets from the Navajo Churro sheep enclosure at the Santa Barbara Zoo (Santa Barbara, CA). Fresh fecal material was returned to the laboratory within 2 h of collection, ground, and suspended into culture Medium C [37]. Resuspended fecal material was diluted in a 10-fold series and 1 mL aliquots of the higher dilutions were used to inoculate anaerobic Hungate tubes containing Medium C and sterilized, 4-mm-milled reed canary grass. Cultures that demonstrated fungal growth and the absence of bacterial contamination were sustained on reed canary grass through routine anaerobic transfers into antibiotic containing culture media. Axenic cultures were obtained using roll tubes (25-mL serum tubes coated with 5 mL of solidified Medium C containing 2% agar and 0.5% cellobiose as the sole carbon source) inoculated with 0.1 mL of actively growing culture. Inoculated roll tubes were incubated for 2–3 days, after which isolated single colonies were selected by cutting them out of the agar and transferring to grow on reed canary grass in fresh, anaerobic liquid culture tubes. This procedure was performed in a Styrofoam box under a constant flow of CO_2_ to maintain anaerobic conditions. This process of colony selection, picking, and culture was completed three times for each strain of gut fungus to ensure selection of a single, isolated strain. These axenic cultures were routinely grown in 10-mL batch cultures of Medium C containing ground reed canary grass (4-mm particle size) in 15-mL anaerobic Hungate tubes. The antibacterial antibiotic was withdrawn from culture media after the single colony isolation process, once it was absolutely certain that cultures did not contain contaminating bacteria. Cultures were routinely transferred to new media every 3–5 days to continue growth. Cultures were also stored cryogenically, as described by Solomon, Henske et al. [38].

### Phylogenetic analysis of isolated fungi

Phylogenetic analysis was completed by sequencing the internal transcribed spacer (ITS) region for each of the isolated fungi. ITS sequences were PCR amplified using the previously described JB206 and JB205 primers [20]. The amplified DNA was sequenced and the ITS1 region was primarily employed in phylogenetic analysis. ITS1 or full ITS sequences were obtained for other anaerobic gut fungi across all known genera from sequences deposited in GenBank [29, 39]. The phylogenetic tree was created using Molecular Evolutionary Genetic Analysis (MEGA) software version 6.0 [40]. Sequences were aligned using the Clustal Omega multiple sequence alignment method [41, 42], and the alignment was used to construct phylogeny using the maximum parsimony method. To test the confidence of the phylogeny, a bootstrap method was used with 1000 replications. Trees were edited for display using the Interactive Tree of Life [43].

### Helium ion microscopy

Helium Ion Microscopy was performed as described in Henske et al. [33]. Briefly fungal cultures were chemically fixed with 2% glutaraldehyde (Sigma Aldrich) and dehydrated through step-gradients from 0 to 70% ethanol. The biomass was then washed with 100% ethanol and dried using critical point drying with an Autosamdri-815 (Tousimis, Rockville, MD) and CO_2_ as a transitional fluid. Dried samples were sputter-coated with conductive carbon and secondary electron images were obtained with an Orion helium ion microscope (Carl Zeiss Microscopy, Peabody, MA).

### Growth analysis of *C. churrovis*

Growth curves of axenic fungal cultures were generated from Medium C grown cultures by measuring the pressure of fermentation gases during growth, which is a precise, indirect measure of fungal proliferation [23]. Soluble substrates, glucose, fructose, galactose, xylose, arabinose, maltose, cellobiose, and sucrose were dissolved in water and sterile filtered. They were added to autoclaved Medium C to a final concentration of 5 g L^−1^. Carboxymethyl cellulose, Avicel^®^, Sigmacell (Sigma Aldrich), xylan (from corn stover, TCI Chemicals, Portland, OR), reed canary grass, corn stover, switchgrass, and alfalfa stems (USDA-ARS Research Center, Madison, WI) were added to a concentration of 10 g L^−1^ prior to autoclaving media. Pressure measurements were taken five times daily for 10 days. Effective net-specific growth rates were determined from the slope of pressure accumulation data plotted against fermentation time of 3 × replicate cultures during the phase of exponential gas accumulation.

### RNA isolation for transcriptome acquisition

RNA was isolated from growing fungal cultures during the exponential growth phase using the Qiagen RNeasy Mini Kit (Qiagen, Valencia, CA), as previously described using the plant and fungi protocol with liquid nitrogen grinding and on-column DNase Digest [5]. RNA was isolated from cultures grown on glucose, fructose, cellobiose, cellulose, and reed canary grass. The RNA quality was determined through measurement on an Agilent Tapestation 2200 (Agilent, Santa Clara, CA) to obtain RINe scores and the minimum RINe score for samples used in transcriptome acquisition was 8.9. The total RNA quantity was determined by using Qubit Fluorometric Quantitation (Qubit, New York, NY) using the high sensitivity RNA reagents.

### RNA sequencing and transcriptome assembly

RNA was pooled prior to generation of the sequencing library using equal quantities of total RNA from each growth condition. After pooling libraries were created using an Illumina Truseq Stranded mRNA library prep kit (Illumina Inc., San Diego, CA) following the kit protocol. The transcriptome was sequenced using the UCSB Biological Nanostructures Laboratory core sequencing facility’s Illumina NextSeq. Coverage greater than 500 × was achieved and assembled and the transcriptome was assembled de novo using Trinity [27].

### Transcriptome annotation and analysis

The transcriptome of *C. churrovis* was annotated as previously described using a combination of NCBI Blast, InterPro, Gene Ontology, and ortholog annotations [5]. Blast annotation was completed using the NCBI standalone blast application to perform blastx against the NCBI non-redundant database downloaded on 11/25/2015 [29] with an E-value cutoff of 10^−3^. Transcripts were then analyzed for protein domains using the BLAST2GO package [44] for alignment to sequences in the EMBL-EBI InterPro database before gene ontology [45] terms and enzyme commission [46] numbers were assigned. Antisense RNA (asRNA) sequences were identified based on the strand specificity of the library and orientation of alignments to BLAST hits. All transcripts were examined for orthology by comparing all possible open reading frames to the OrthoMCL database [47].

### Gut fungal transcriptome sequence comparisons

Alignment of full gut fungal transcriptomes was completed using the standalone BLAST tool kit [29]. BLAST databases were created from full transcriptome fasta files using the “makeblastdb” function. The blastn function was then used to align transcriptome fasta files to transcriptome databases. For identification of scaffoldin transcripts, amino acid sequences of four scaffoldin transcripts from *Piromyces finnis* [13] were aligned to the *C. churrovis* transcriptome nucleotide database using tblastn which aligns the amino acid sequences to the translated (in all frames) sequences in the database.

### Cellulase activity assays

Fungal enzymatic activity on Carboxymethyl Cellulose (CMC) (Sigma Aldrich), xylan (from corn stover, TCI Chemicals, Portland, OR), and pectin (from citrus fruits, MP biomedicals) was measured essentially as described previously [5]. Briefly, 50 µL of a 2% substrate solution in citrate–phosphate buffer (pH 6.5) was combined with 30 µL of the cellulosome fraction or supernatant. The reducing sugar concentration was measured by adding 60 µL of DNS to 30 µL of reaction and then heating the solution at 95 °C for 5 min. 36 µL of the completed DNS reaction were transferred to 160 µL of water and the absorbance was measured at 540 nm. Rates were calculated by comparing to a standard curve constructed from glucose, and by subtracting a blank measurement where blank buffer was added to the substrate. In all cases, samples were performed in triplicate, and all values were normalized by total protein as measured by a BCA protein assay kit (Pierce Biotechnology, Rockford, IL). Cellulosome fractions were prepared as previously described using cellulose to precipitate cellulosome complexes from fungal supernatant after growth for 6 days on reed canary grass in Medium C [5, 36].

## Additional file

**Additional file 1: Table S1.** Alignment results for transcriptomes of *A. robustus*, *N. californiae*, and *P. finnis* [5]. Shown are # of transcripts (% of transcriptome) in the transcriptome of the fungus listed on the left side successfully aligned to the transcriptome of the fungus listed across the top row using blastn analysis. **Table S2.** Alignment of scaffoldin amino acid sequences from *P. finnis* [13] to the transcriptome of *C. churrovis* by tblastn identifies scaffoldin transcripts. Only results with an alignment E value of 0 are shown. **Figure S1.** ITS1 Phylogeny of *Caecomyces* strains shows *C. churrovis* is significantly different compared to other strains. ITS1 phylogeny of only Caecomyces fungal strains identified a clear separation of *C. churrovis* from other isolated strains. **Figure S2.** Full ITS Phylogeny confirms observations about *C. churrovis*. Phylogeny of ITS1-5.8S-ITS2 regions confirmed the observations that *C. churrovis* represents a new species in the *Caecomyces* genus. **Figure S3.** Catabolic pathways for biomass derived sugars were reconstructed using transcriptome annotations. Enzyme commission numbers and BLAST alignments were used to identify complete sugar pathways present in the transcriptome of *C. churrovis*. This analysis revealed catabolic routes for glucose, xylose, and fructose, but not mannose, sucrose, and arabinose. Catabolism of α-d-galactose was identified using BLAST annotations, but not EC numbers. **Figure S4.** The secretomes of anaerobic gut fungi display free enzymes and multi enzymes complexes (cellulosomes). The same amount of secreted proteins (determined by BCA assay) of *P. finnis* (F), *N. californiae* (G1), *A. robustus* (S4) and *C. churrovis* (C) were loaded on Native (A) and SDS (B) PAGE. While the Native PAGE (stained by silver staining) shows strong bands indicative of cellulosomes around 1200 kDa, the SDS PAGE (stained by SYPRO Ruby) shows many bands in dissociated cellulosome complexes.

## References

[1] Theodorou MK, Mennim G, Davies DR, Zhu WY, Trinci AP, Brookman JL (1996). Anaerobic fungi in the digestive tract of mammalian herbivores and their potential for exploitation. Proc Nutr Soc.

[2] Liggenstoffer AS, Youssef NH, Couger MB, Elshahed MS (2010). Phylogenetic diversity and community structure of anaerobic gut fungi (phylum Neocallimastigomycota) in ruminant and non-ruminant herbivores. ISME J.

[3] Haitjema CH, Solomon KV, Henske JK, Theodorou MK, O’Malley MA (2014). Anaerobic gut fungi: advances in isolation, culture, and cellulolytic enzyme discovery for biofuel production. Biotechnol Bioeng.

[4] Ljungdahl LG (2008). The cellulase/hemicellulase system of the anaerobic fungus *Orpinomyces* PC-2 and aspects of its applied use. Ann N Y Acad Sci.

[5] Solomon KV, Haitjema CH, Henske JK, Gilmore SP, Borges-Rivera D, Lipzen A (2016). Early-branching gut fungi possess a large, comprehensive array of biomass-degrading enzymes. Science.

[6] Gilmore SP, Henske JK, O’Malley MA (2015). Driving biomass breakdown through engineered cellulosomes. Bioengineered.

[7] Mountfort DO, Orpin CG (1994). Anaerobic fungi: biology, ecology, and function.

[8] Dagar SS, Kumar S, Griffith GW, Edwards JE, Callaghan TM, Singh R, Nagpal AK, Puniya AK (2015). A new anaerobic fungus (*Oontomyces anksri* gen. nov., sp. nov.) from the digestive tract of the Indian camel (*Camelus dromedarius*). Fungal Biol..

[9] Griffith GW, Callaghan TM, Podmirseg SM, Hohlweck D, Edwards JE, Puniya AK, Dagar SS (2015). *Buwchfawromyces eastonii* gen. nov., sp. nov.: a new anaerobic fungus (Neocallimastigomycota) isolated from buffalo faeces. MycoKeys.

[10] Hanafy RA, Elshahed MS, Liggenstoffer AS, Griffith GW, Youssef NH (2017). *Pecoramyces ruminantium*, gen. nov., sp. nov., an anaerobic gut fungus from the feces of cattle and sheep. Mycologia.

[11] Ho YW, Abdullah N, Jalaludin S (1988). Penetrating structures of anaerobic rumen fungi in cattle and swamp buffalo. Microbiology.

[12] Ozkose E, Thomas BJ, Davies DR, Griffith GW, Theodorou MK (2001). *Cyllamyces aberensis* gen. nov. sp. nov., a new anaerobic gut fungus with branched sporangiophores isolated from cattle. Can J Bot.

[13] Haitjema CH, Gilmore SP, Henske JK, Solomon KV, Groot RD, Kuo A, Mondo SJ, Salamov AA, Labutti K, Zhao Z (2017). A parts list for fungal cellulosomes revealed by comparative genomics. Nat Microbiol.

[14] Youssef NH, Couger MB, Struchtemeyer CG, Liggenstoffer AS, Prade RA, Najar FZ (2013). The genome of the anaerobic fungus Orpinomyces sp. strain C1A reveals the unique evolutionary history of a remarkable plant biomass degrader. Appl Environ Microbiol.

[15] Chen Y-C, Tsai S-D, Cheng H-L, Chien C-Y, Hu C-Y, Cheng T-Y (2017). *Caecomyces sympodialis* sp. nov., a new rumen fungus isolated from Bos indicus. Mycologia.

[16] Wubah DA, Fuller MS, Akin DE (1991). Studies on *Caecomyces communis*: morphology and development. Mycologia.

[17] Hodrova B, Kopecny J, Kas J (1998). Cellulolytic enzymes of rumen anaerobic fungi *Orpinomyces joyonii* and *Caecomyces communis*. Res Microbiol.

[18] Schoch CL, Seifert KA, Huhndorf S, Robert V, Spouge JL, Levesque CA, Chen W, Fungal Barcoding C (2012). Fungal Barcoding Consortium Author L: nuclear ribosomal internal transcribed spacer (ITS) region as a universal DNA barcode marker for fungi. Proc Natl Acad Sci USA.

[19] Brookman JL, Mennim G, Trincia P, Theodorou MK, Tuckwell DS (2000). Identification and characterization of anaerobic gut fungi using molecular methodologies based on ribosomal ITS1 and 18S rRNA. Microbiology.

[20] Tuckwell DS, Nicholson MJ, McSweeney CS, Theodorou MK, Brookman JL (2005). The rapid assignment of ruminal fungi to presumptive genera using ITS1 and ITS2 RNA secondary structures to produce group-specific fingerprints. Microbiology.

[21] Nicholson MJ, McSweeney CS, Mackie RI, Brookman JL, Theodorou MK (2010). Diversity of anaerobic gut fungal populations analysed using ribosomal ITS1 sequences in faeces of wild and domesticated herbivores. Anaerobe.

[22] Li GJ, Hyde KD, Zhao RL, Hongsanan S, Abdel-Aziz FA, Abdel-Wahab MA (2016). Fungal diversity notes 253–366: taxonomic and phylogenetic contributions to fungal taxa. Fungal Divers.

[23] Theodorou MK, Davies DR, Nielsen BB, Lawrence MIG, Trinci APJ (1995). Determination of growth of anaerobic fungi on soluble and cellulosic substrates using a pressure transducer. Microbiology.

[24] Theodorou MK, Williams B, Dhanoa MS, McAllan AB, France J (1994). A simple gas production method using a pressure transducer to determine the fermentation kinetics of ruminant feeds. Anim Feed Sci Technol.

[25] Dien B, Jung H, Vogel K, Casler M, Lamb J, Iten L, Mitchell R, Sarath G (2006). Chemical composition and response to dilute-acid pretreatment and enzymatic saccharification of alfalfa, reed canarygrass, and switchgrass. Biomass Bioenerg.

[26] Pordesimo LO, Hames BR, Sokhansanj S, Edens WC (2005). Variation in corn stover composition and energy content with crop maturity. Biomass Bioenerg.

[27] Grabherr MG, Haas BJ, Yassour M, Levin JZ, Thompson DA, Amit I, Adiconis X, Fan L, Raychowdhury R, Zeng Q (2011). Full-length transcriptome assembly from RNA-Seq data without a reference genome. Nat Biotechnol.

[28] Martin J, Bruno VM, Fang Z, Meng X, Blow M, Zhang T, Sherlock G, Snyder M, Wang Z (2010). Rnnotator: an automated de novo transcriptome assembly pipeline from stranded RNA-Seq reads. BMC Genom.

[29] NCBI Resource Coordinators (2016). Database resources of the national center for biotechnology information. Nucleic Acids Res.

[30] Finn RD, Attwood TK, Babbitt PC, Bateman A, Bork P, Bridge AJ, Chang HY, Dosztanyi Z, El-Gebali S, Fraser M (2017). InterPro in 2017-beyond protein family and domain annotations. Nucleic Acids Res.

[31] Nicholson MJ, Theodorou MK, Brookman JL (2005). Molecular analysis of the anaerobic rumen fungus Orpinomyces—insights into an AT-rich genome. Microbiology.

[32] Brownlee AG (1989). Remarkably AT-rich genomic DNA from the anaerobic fungus Neocallimastix. Nucleic Acids Res.

[33] Henske JK, Wilken SE, Solomon KV, Smallwood CR, Shutthanandan V, Evans JE, Theodorou MK, O'Malley MA (2017). Metabolic characterization of anaerobic fungi provides a path forward for two-stage bioprocessing of crude lignocellulose. Biotechnol Bioeng.

[34] Mewis K, Lenfant N, Lombard V, Henrissat B (2016). Dividing the large glycoside hydrolase family 43 into subfamilies: a motivation for detailed enzyme characterization. Appl Environ Microbiol.

[35] Biely P (2012). Microbial carbohydrate esterases deacetylating plant polysaccharides. Biotechnol Adv.

[36] Ali BR, Zhou L, Graves FM, Freedman RB, Black GW, Gilbert HJ, Hazelwood GP (1995). Cellulases and hemicellulases of the anaerobic fungus Piromyces constitute a multiprotein cellulose-binding complex and are encoded by multigene families. FEMS Microbiol Lett.

[37] Theodorou MK, Brookman J, Trinci APJ, Makkar HPS, McSweeney CS (2005). Anaerobic Fungi. Methods in gut microbial ecology for ruminants.

[38] Solomon KV, Henske JK, Theodorou MK, O’Malley MA (2016). Robust and effective methodologies for cryopreservation and DNA extraction from anaerobic gut fungi. Anaerobe.

[39] Benson DA, Cavanaugh M, Clark K, Karsch-Mizrachi I, Lipman DJ, Ostell J (2013). GenBank. Nucleic Acids Res.

[40] Tamura K, Stecher G, Peterson D, Filipski A, Kumar S (2013). MEGA6: Molecular Evolutionary Genetics Analysis version 6.0. Mol Biol Evol.

[41] Chenna R (2003). Multiple sequence alignment with the Clustal series of programs. Nucleic Acids Res.

[42] Sievers F, Wilm A, Dineen D, Gibson TJ, Karplus K, Li W, Lopez R, McWilliam H, Remmert M, Soding J (2011). Fast, scalable generation of high-quality protein multiple sequence alignments using clustal omega. Mol Syst Biol.

[43] Letunic I, Bork P (2016). Interactive tree of life (iTOL) v3: an online tool for the display and annotation of phylogenetic and other trees. Nucleic Acids Res.

[44] Conesa A, Gotz S, Garcia-Gomez JM, Terol J, Talon M, Robles M (2005). Blast2GO: a universal tool for annotation, visualization and analysis in functional genomics research. Bioinformatics.

[45] Ashburner M, Ball CA, Blake JA, Botstein D, Butler H, Cherry JM, Davis AP, Dolinski K, Dwight SS, Eppig JT (2000). Gene ontology: tool for the unification of biology. The Gene Ontology Consortium. Nat Genet.

[46] Bairoch A (2000). The ENZYME database in 2000. Nucleic Acids Res.

[47] Li L, Stoeckert CJJ, Roos DS (2003). OrthoMCL: identification of ortholog groups for eukaryotic genomes. Genome Res.

