# Supplementary material for: Transcriptomic characterization of Caecomyces churrovis: a novel, non-rhizoid-forming lignocellulolytic anaerobic fungus
Source: Biotechnol Biofuels. 2017 Dec 20;10:305. doi: 10.1186/s13068-017-0997-4 (PMC5737911; doi:10.1186/s13068-017-0997-4)
Supplement: Supplementary file 1 — Additional file 1: Table S1. Alignment results for transcriptomes of A. robustus, N. californiae, and P. finnis [5]. Shown are # of transcripts (% of transcriptome) in the transcriptome of the fungus listed on the left side successfully aligned to the transcriptome of the fungus listed across the top row using blastn analysis. Table S2. Alignment of scaffoldin amino acid sequences from P. finnis [13] to the transcriptome of C. churrovis by tblastn identifies scaffoldin transcripts. Only results with an alignment E value of 0 are shown. Figure S1. ITS1 Phylogeny of Caecomyces strains shows C. churrovis is significantly different compared to other strains. ITS1 phylogeny of only Caecomyces fungal strains identified a clear separation of C. churrovis from other isolated strains. Figure S2. Full ITS Phylogeny confirms observations about C. churrovis. Phylogeny of ITS1-5.8S-ITS2 regions confirmed the observations that C. churrovis represents a new species in the Caecomyces genus. Figure S3. Catabolic pathways for biomass derived sugars were reconstructed using transcriptome annotations. Enzyme commission numbers and BLAST alignments were used to identify complete sugar pathways present in the transcriptome of C. churrovis. This analysis revealed catabolic routes for glucose, xylose, and fructose, but not mannose, sucrose, and arabinose. Catabolism of α-d-galactose was identified using BLAST annotations, but not EC numbers. Figure S4. The secretomes of anaerobic gut fungi display free enzymes and multi enzymes complexes (cellulosomes). The same amount of secreted proteins (determined by BCA assay) of P. finnis (F), N. californiae (G1), A. robustus (S4) and C. churrovis (C) were loaded on Native (A) and SDS (B) PAGE. While the Native PAGE (stained by silver staining) shows strong bands indicative of cellulosomes around 1200 kDa, the SDS PAGE (stained by SYPRO Ruby) shows many bands in dissociated cellulosome complexes. [file 13068_2017_997_MOESM1_ESM.zip › supplementary data/Caecomyces_BiotechBiofuels_Supplement_revision.docx]

Supplemental Materials for

**Transcriptomic characterization of *Caecomyces churrovis*: a novel, non-rhizoid forming lignocellulolytic anaerobic fungus**

John K. Henske^1^, Sean P. Gilmore^1^, Doriv Knop^1^, Francis J. Cunningham^1^, Jessica Sexton^1^, Chuck R. Smallwood^2^, Vaithiyalingam Shutthanandan^2^, James E. Evans^2^, Michael K. Theodorou^3^, Michelle A. O’Malley^1ǂ^

^1^Department of Chemical Engineering, University of California, Santa Barbara, CA 93106, USA

^2^Environmental Molecular Sciences Laboratory, Pacific Northwest National Laboratory, Richland, WA 99354, USA

^3^Animal Production, Welfare and Veterinary Sciences, Harper Adams University, Newport, Shropshire, TF10 8NB, UK

^ǂ^Corresponding Author. E-mail: [momalley@engineering.ucsb.edu](mailto:momalley@engineering.ucsb.edu)

**Table S1**. Alignment results for transcriptomes of *A. robustus*, *N. californiae*, and *P. finnis* (Solomon, Haitjema et al. 2016). Shown are # of transcripts (% of transcriptome) in the transcriptome of the fungus listed on the left side successfully aligned to the transcriptome of the fungus listed across the top row using blastn analysis.

|  | *A. Robustus* | *N. californiae* | *P. finnis* |
| --- | --- | --- | --- |
| *A. Robustus* | X | 8,446 (49.31%) | 6,971 (40.70%) |
| *N. californiae* | 12,814 (43.22%) | X | 10,813 (36.47%) |
| *P. finnis* | 7,146 (42.02%) | 7,297 (42.90%) | X |

**Table S2**. Alignment of scaffoldin amino acid sequences from *P. finnis* (Haitjema, Gilmore et al. 2017) to the transcriptome of *C. churrovis* by tblastn identifies scaffoldin transcripts. Only results with an alignment E value of 0 are shown.

| *P. finnis* Scaffoldin Transcript | *P. finnis* transcript length (nt) | *C. churrovis* Transcript | *C. churrovis* transcript length (nt) | Alignment Bit Score | E-value | Hit Length |
| --- | --- | --- | --- | --- | --- | --- |
| Locus833v1rpkm50.85 | 17631 | TR4850\|c0_g1_i1 | 18495 | 2188.69 | 0 | 6143 |
|  |  | TR4850\|c0_g1_i2 | 18497 | 1969.9 | 0 | 5200 |
| Locus5834v3rpkm1.66 | 5013 | TR12670\|c2_g1_i2 | 3676 | 1503.42 | 0 | 1225 |
|  |  | TR12670\|c2_g1_i1 | 3694 | 1490.71 | 0 | 1225 |
|  |  | TR2884\|c4_g1_i1 | 4019 | 1275.00 | 0 | 1271 |
| Locus1577v1rpkm21.29 | 4230 | TR119\|c3_g1_i1 | 8088 | 960.67 | 0 | 1267 |
|  |  | TR119\|c4_g1_i1 | 3259 | 927.93 | 0 | 1080 |
|  |  | TR118\|c3_g1_i1 | 7967 | 855.90 | 0 | 1257 |
|  |  | TR118\|c1_g3_i1 | 4423 | 843.57 | 0 | 1275 |
|  |  | TR10527\|c0_g1_i1 | 4233 | 805.82 | 0 | 1242 |
|  |  | TR7370\|c0_g1_i1 | 4343 | 803.90 | 0 | 1312 |
|  |  | TR1820\|c0_g2_i1 | 4954 | 787.72 | 0 | 1219 |
|  |  | TR118\|c1_g2_i1 | 4587 | 783.48 | 0 | 1279 |
|  |  | TR12725\|c1_g1_i1 | 6611 | 778.86 | 0 | 1400 |
|  |  | TR13610\|c0_g2_i1 | 3971 | 774.24 | 0 | 1184 |
|  |  | TR1958\|c0_g1_i1 | 4205 | 764.61 | 0 | 1300 |
|  |  | TR1958\|c0_g2_i1 | 3319 | 747.66 | 0 | 1095 |
|  |  | TR18143\|c0_g1_i1 | 4704 | 709.52 | 0 | 1395 |
|  |  | TR12054\|c0_g1_i1 | 4227 | 702.21 | 0 | 1338 |
|  |  | TR119\|c5_g1_i1 | 1271 | 615.92 | 0 | 366 |
|  |  | TR12670\|c2_g1_i2 | 3676 | 605.52 | 0 | 1219 |
|  |  | TR12670\|c2_g1_i1 | 3694 | 588.19 | 0 | 1225 |
| Locus5976v1rpkm2.95 | 7671 | TR119\|c3_g1_i1 | 8088 | 1826.99 | 0 | 1896 |
|  |  | TR119\|c2_g1_i1 | 5221 | 1541.55 | 0 | 1793 |
|  |  | TR118\|c3_g1_i1 | 7967 | 1484.55 | 0 | 1708 |
|  |  | TR1820\|c0_g2_i1 | 4954 | 1403.27 | 0 | 1659 |
|  |  | TR12725\|c1_g1_i1 | 6611 | 1355.50 | 0 | 1574 |
|  |  | TR1958\|c0_g1_i1 | 4205 | 1306.58 | 0 | 1364 |
|  |  | TR10527\|c0_g1_i1 | 4233 | 1112.44 | 0 | 1387 |
|  |  | TR1958\|c0_g2_i1 | 3319 | 1073.92 | 0 | 1080 |
|  |  | TR118\|c1_g3_i1 | 4423 | 990.33 | 0 | 1277 |
|  |  | TR7370\|c0_g1_i1 | 4343 | 805.05 | 0 | 1297 |
|  |  | TR118\|c1_g2_i1 | 4587 | 786.95 | 0 | 1281 |
|  |  | TR13610\|c0_g2_i1 | 3971 | 763.07 | 0 | 1274 |
|  |  | TR18143\|c0_g1_i1 | 4704 | 734.56 | 0 | 1342 |
|  |  | TR12054\|c0_g1_i1 | 4227 | 718.38 | 0 | 1341 |
|  |  | TR119\|c4_g1_i1 | 3259 | 647.89 | 0 | 910 |


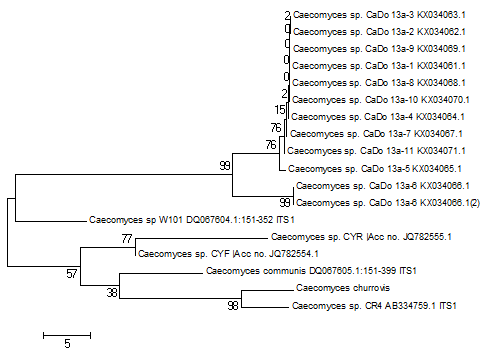


**Figure S1. ITS1 Phylogeny of *Caecomyces* strains shows *C. churrovis* is significantly different compared to other strains.** ITS1 phylogeny of only Caecomyces fungal strains identified a clear separation of *C. churrovis* from other isolated strains.


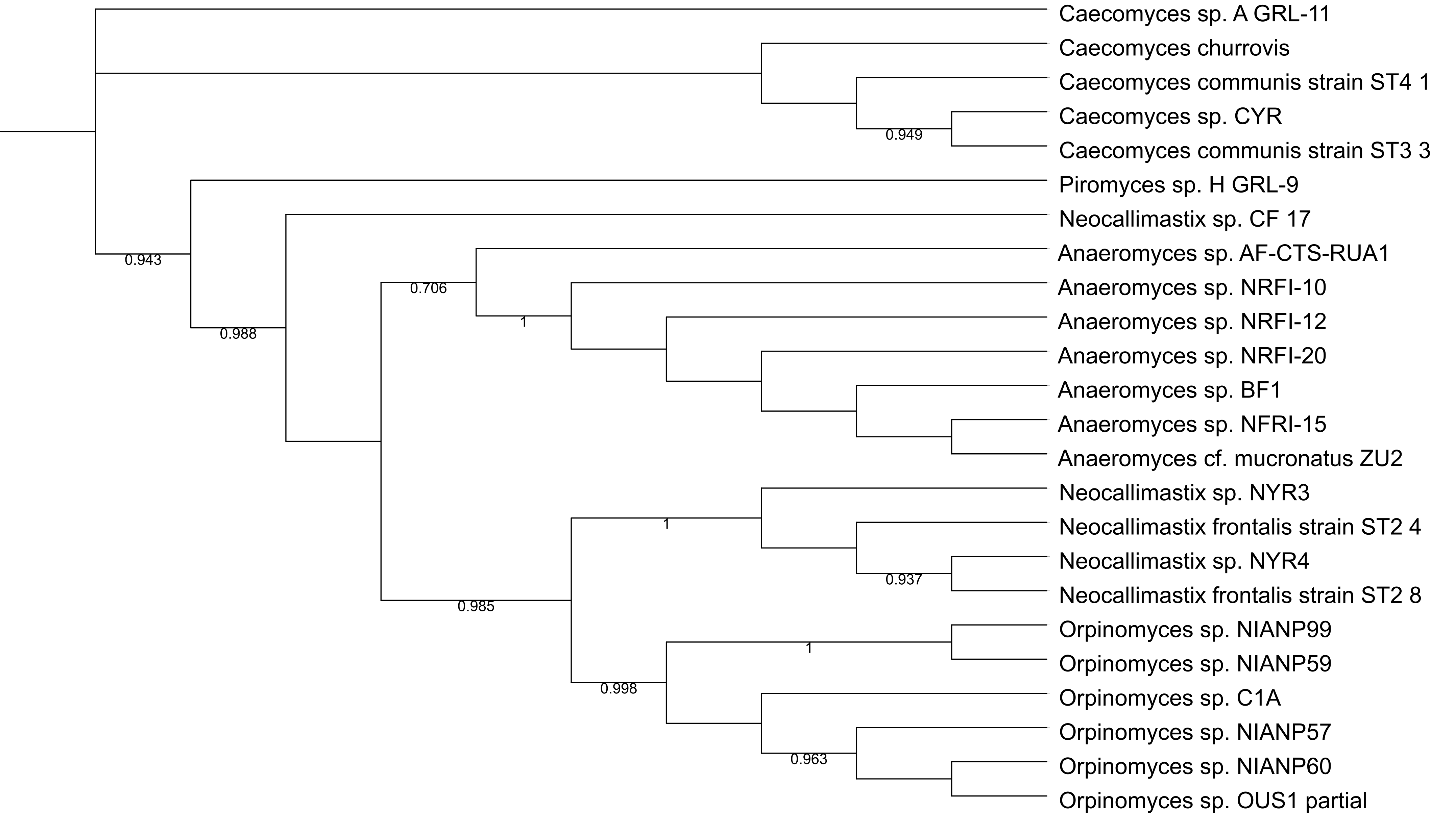


**Figure S2. Full ITS Phylogeny confirms observations about *C. churrovis*.** Phylogeny of ITS1-5.8S-ITS2 regions confirmed the observations that *C. churrovis* representes a new species in the *Caecomyces* genus.


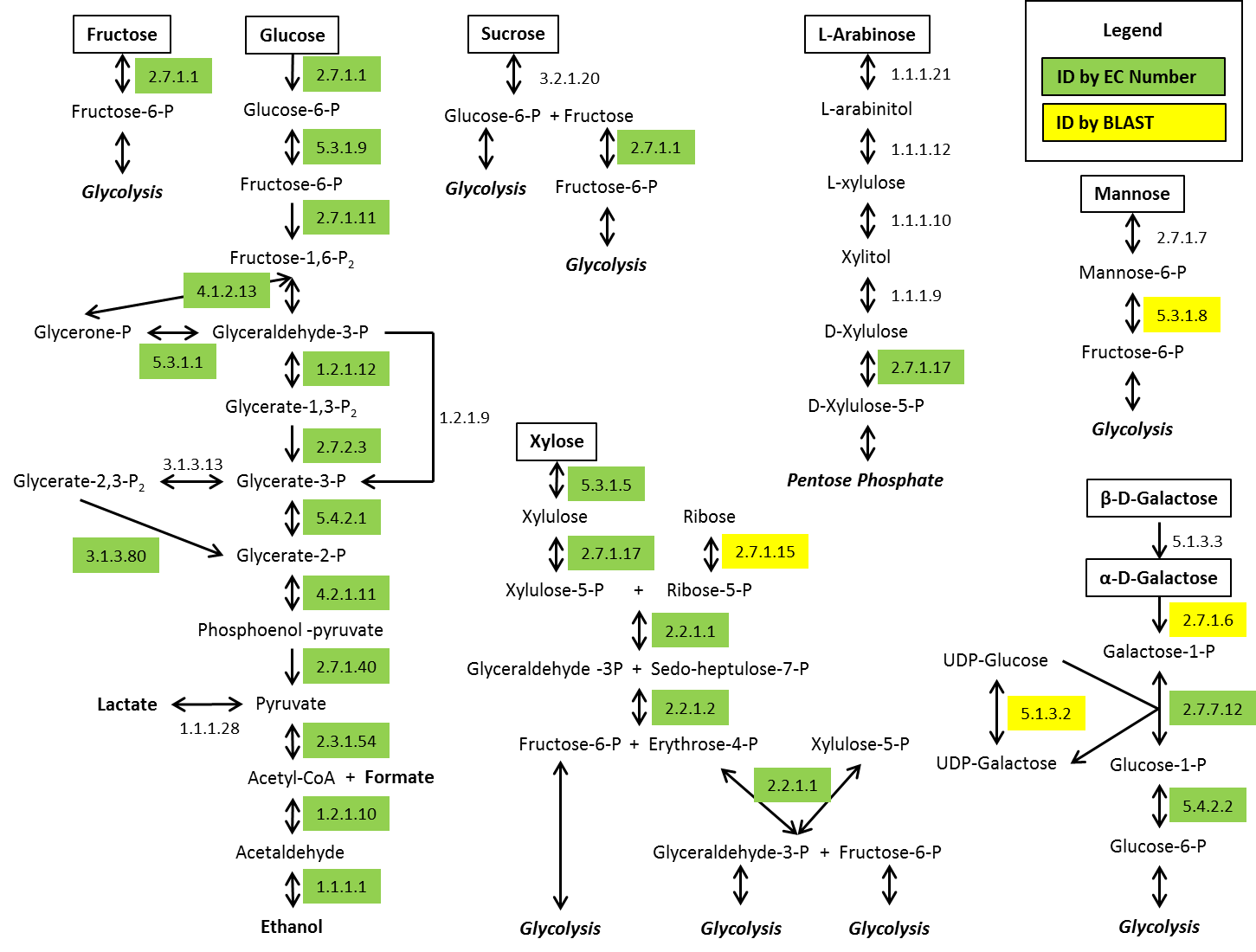


**Figure S3. Catabolic pathways for biomass derived sugars were reconstructed using transcriptome annotations.** Enzyme commission numbers and BLAST alignments were used to identify complete sugar pathways present in the transcriptome of *C. churrovis*. This analysis revealed catabolic routes for glucose, xylose, and fructose, but not mannose, sucrose, and arabinose. Catabolism of α-D-galactose was identified using BLAST annotations, but not EC numbers.

**
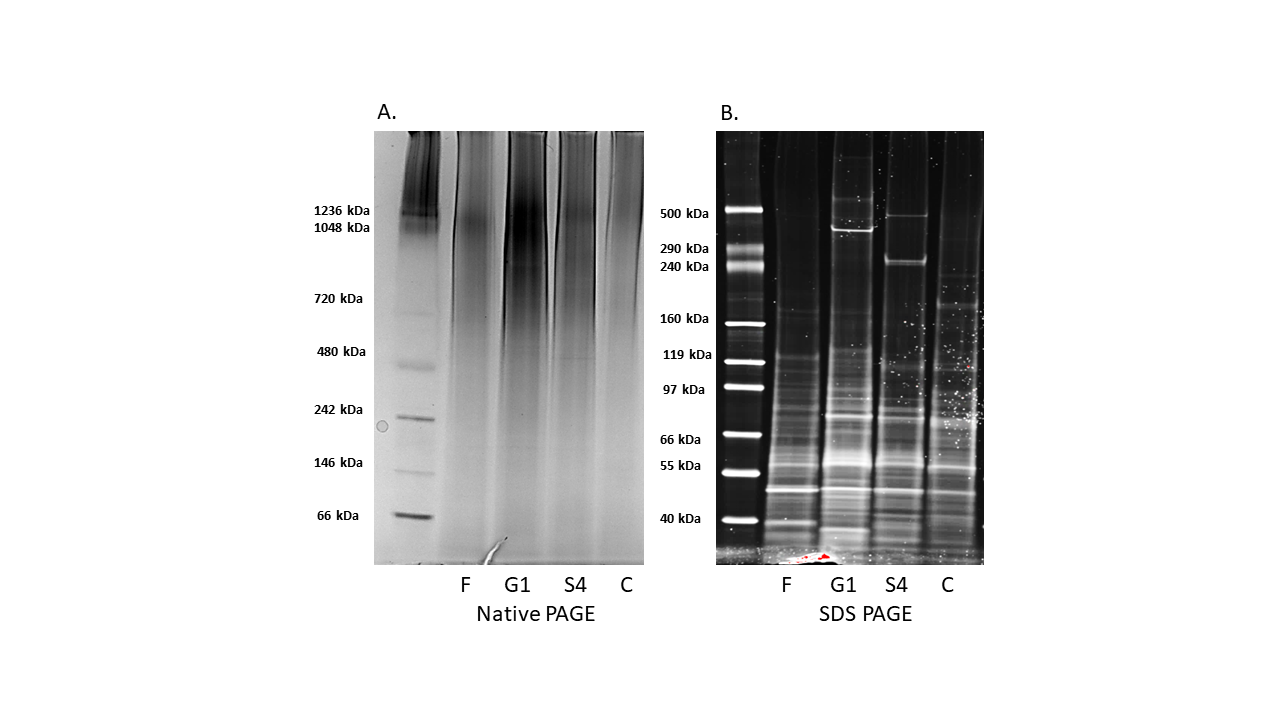
**

**Figure S4. The secretomes of anaerobic gut fungi display free enzymes and multi enzymes complexes (cellulosomes).** The same amount of secreted proteins (determined by BCA assay) of *P. finnis* (F), *N. californiae* (G1), *A. robustus* (S4) and *C. churrovis* (C) were loaded on Native (A) and SDS (B) PAGE. While the Native PAGE (stained by silver staining) shows strong bands indicative of cellulosomes around 1200kDa, the SDS PAGE (stained by SYPRO Ruby) shows many bands in dissociated cellulosome complexes.

**Additional Materials**

Data S1 – *Caecomyces churrovis* transcriptome (.fasta file)

Data S2 – *C. churrovis* transcriptome annotation table (tab-delimited text file)

Data S3 – ITS1 Sequences used for phylogenetic analysis (.fasta file)

Data S4 - Full ITS Region for *C. churrovis*: ITS1, 5.8S, ITS2, partial 28S

**References**

Haitjema, C. H., S. P. Gilmore, J. K. Henske, K. V. Solomon, R. D. Groot, A. Kuo, S. J. Mondo, A. A. Salamov, K. Labutti, Z. Zhao, J. Chiniquy, K. Barry, H. M. Brewer, S. O. Purvine, A. T. Wright, M. Hainaut, B. Boxma, T. V. Alen, J. H. P. Hackstein, B. Henrissat, S. E. Baker, I. V. Grigoriev and M. A. O. Malley (2017). "A parts list for fungal cellulosomes revealed by comparative genomics." Nature Microbiology **2**. doi:10.1038/nmicrobiol.2017.87

Solomon, K. V., C. H. Haitjema, J. K. Henske, S. P. Gilmore, D. Borges-Rivera and A. Lipzen (2016). "Early

branching gut fungi possess a large, comprehensive array of biomass-degrading enzymes." Science **351**:

1192-1195 (2016).
